# Isolated ectopic cilia in an 11-year-old girl

**DOI:** 10.3205/oc000050

**Published:** 2016-10-12

**Authors:** Hosny Ahmed Zein, M. Tarek A. Moustafa

**Affiliations:** 1 Department of Ophthalmology, Minia University, Minia, Egypt

**Keywords:** eyelids, cilia, pathology

## Abstract

Ectopic cilia (EC) are a very rare condition with only few cases reported in literature. Many associations were seen with ectopic cilia which include distichiasis, choristoma and aberrant lacrimal gland, hypochromic nevus, atopic eczema and others. We are reporting a case of an 11-year-old girl with isolated left upper lid ectopic cilia, which was confirmed by surgical removal and histopathological study.

## Introduction

Ectopic cilia (EC) are a very rare condition with only a few reported cases in the literature [1], [2], [3]. The most common presentations are the abnormal growth of eyelashes on the external, lateral quadrant of the upper eyelid, or the internal, conjunctival surface of the eyelid. There is usually a negative family history, no ocular symptoms but significant cosmetic disfigurement. The ectopic cilia present as a tuft of eyelash follicles on the temporal, upper eyelid. After excision, the pathologic diagnosis is confirmed by the presence of pilosebaceous follicle (PSF) with sebaceous and sweat glands in the specimen.

## Case description

**Figure 1:**
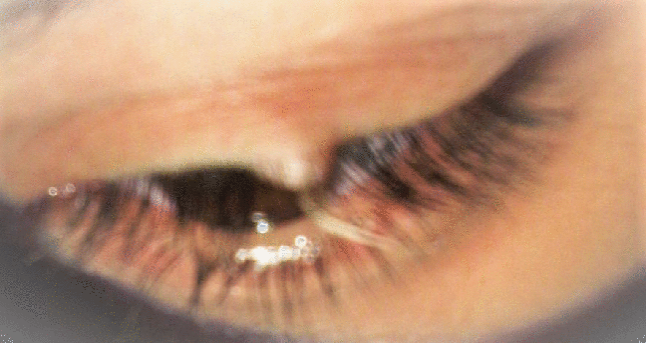
Clinical photograph of ectopic cilia

An 11-year-old girl was seen on September 20 2015 in the ophthalmology outpatient clinic at Minia university hospital, with a chief complaint of a hair tuft projecting between the left upper eyelid lashes (Figure 1 (Fig. 1)). The hair tuft had been present early after birth and epilation had been performed three times. However, the hair tuft recurred within one month after treatment. The ophthalmology examination was normal except for a group of abnormal lashes about 4 mm above the upper lid margin. The patient was otherwise healthy and a dermatological consultation showed no systemic abnormalities with respect to her hair or skin pigmentation. The family history was negative for hair or skin abnormalities. 

**Figure 2:**
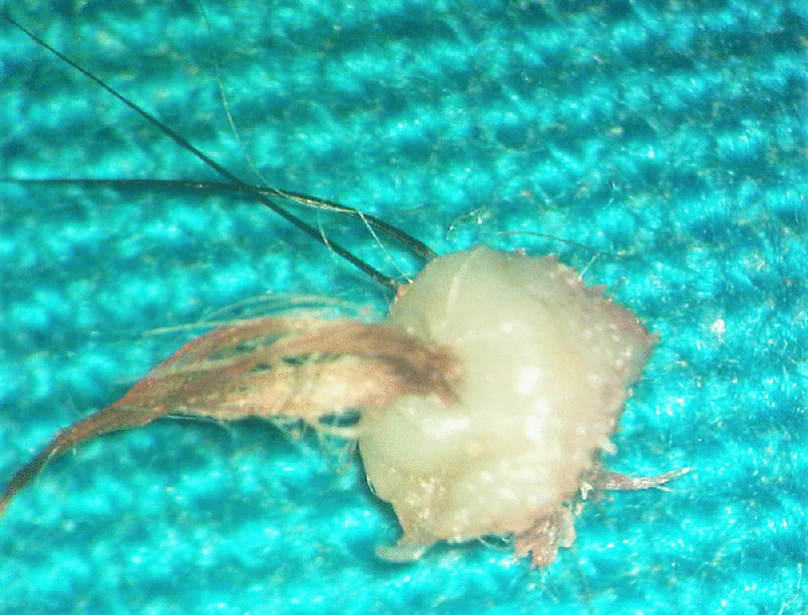
Clinical photograph of the specimen after surgical excision

**Figure 3:**
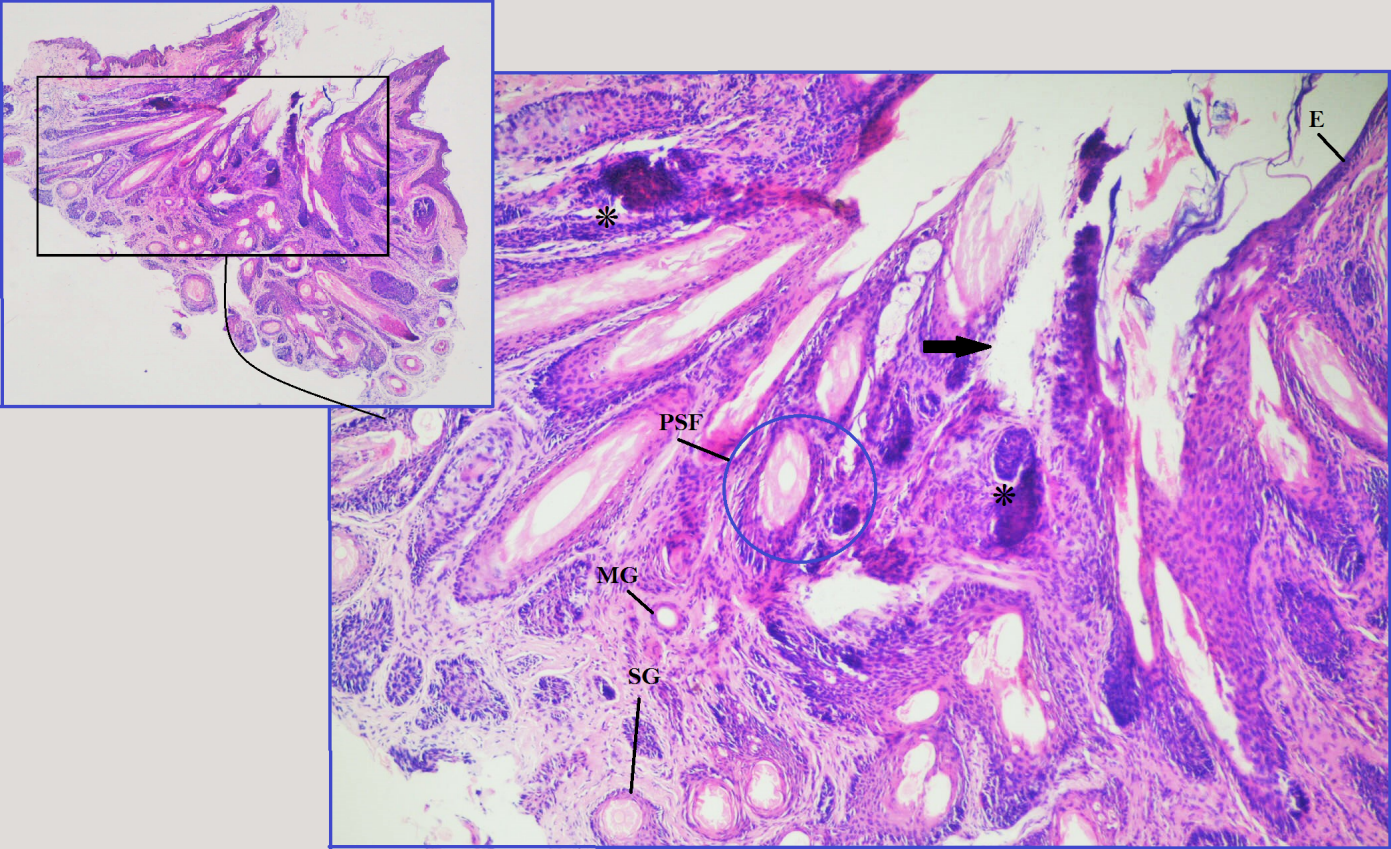
Photomicrograph shows lash follicle (*), its fibrous tract (arrow), epidermis (E), Molls gland (MG), sebaceous gland (SG) and pilosebaceous follicle (PSF).

**Figure 4:**
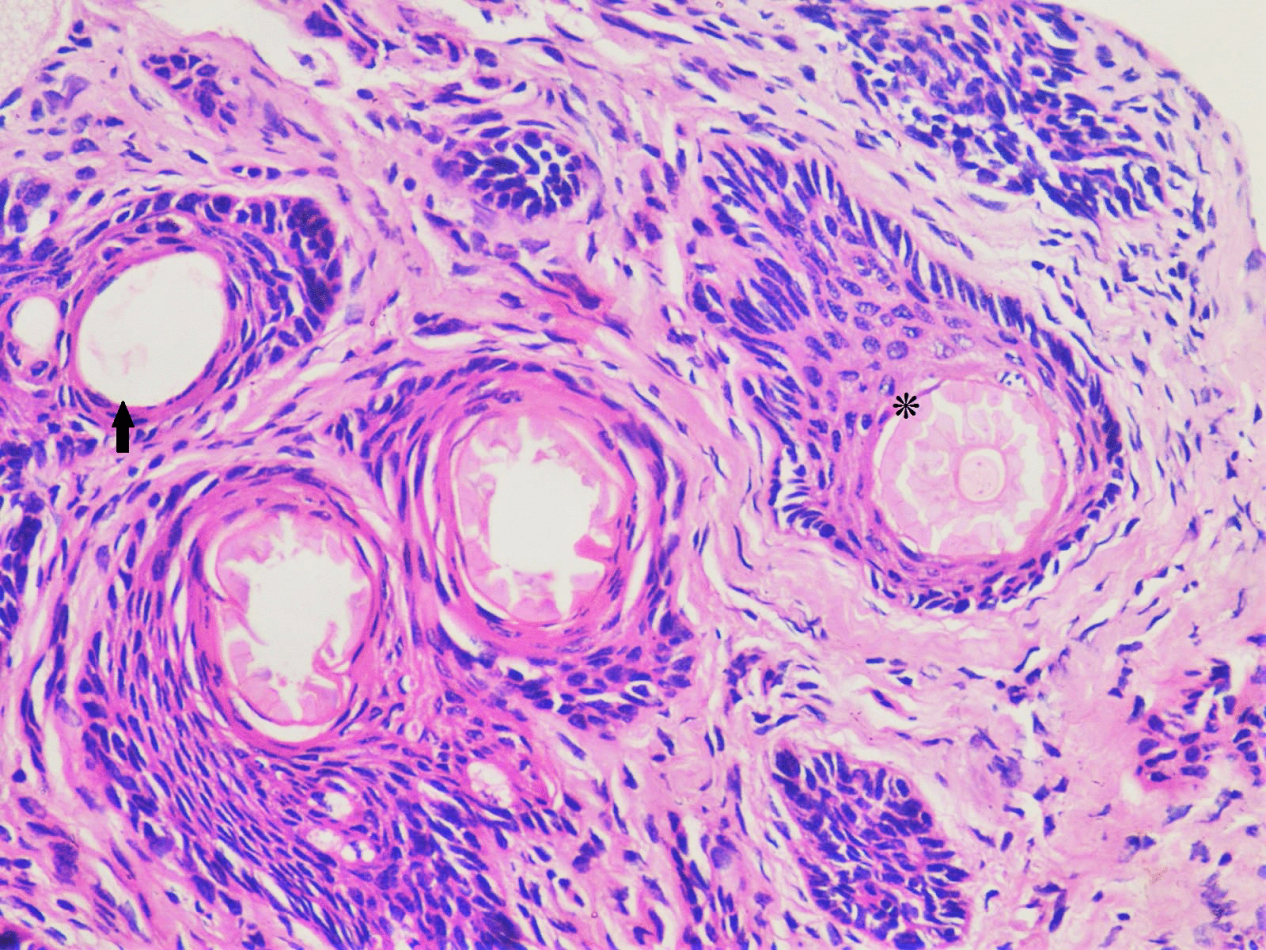
Photomicrograph shows sebaceous gland lobules (*) and epithelial-lined sweat gland (arrow).

After discussing the surgery with her parents, an informed consent was obtained and an excisional biopsy was performed in the operating room under complete aseptic conditions. The left upper eyelid was injected locally with a 1:1 mixture of Xylocaine (2%) and Epinephrine (1:100,000). The tissue specimen was removed as a circular mass with a diameter of 8 mm that included skin, subcutaneous tissue with a centrally situated hair tuft, and some normal lashes located on one side of the abnormal hair tuft (Figure 2 (Fig. 2)). The specimen was sent for histopathology and the report described both sebaceous glands (SG) and epithelial lined apocrine Molls glands (MG) adjacent to subcutaneous pilosebaceous follicle (PSF) (Figure 3 (Fig. 3) and Figure 4 (Fig. 4)). No other glands could be identified. The patient was examined at 1 week, 4 weeks and 3 months postoperatively and no evidence of local recurrence was found.

## Discussion

Ectopic cilia are rarely found in humans with only about 20 cases reported in the literature. The origin of these abnormally situated lashes is not yet clear. Previous reported cases described either congenital ectopic cilia originating from the anterior temporal aspect of the upper tarsal plate [1], [4], [5] or acquired post-inflammatory cilia located in the tarsal conjunctiva [6]. We believe that our case belongs to the first group. 

The ectopic cilia are not usually isolated but are often associated with distichiasis [7], choristoma and aberrant lacrimal glands [8], hypochromic nevus [3], sebum accumulation [9], atopic eczema [10], nail-patella syndrome [11], and a combination of orbital dermoid cyst and sinus tract [2]. However, in our case we could not find any eye-related or systemic abnormalities.

The embryologic origin of the ectopic lashes has been thought to be a deformity of the upper lid glands, with complete or partial replacement of the meibomian glands with skin glands [5]. The anatomical location, as well as the congenital development of Tessier Type 9 facial cleft at the same site support the theory that ectopic cilia is a congenital anomaly, as this site is embryologically related to the watershed area of two angisomes, the superficial temporal artery and the termination of the facial artery [12]. 

## Summary

Ectopic cilia are rare and can present either as an isolated event or in association with other ocular and systemic abnormalities. After surgical removal, the histopathological identification of pilosebaceous follicle, Molls and sebaceous glands are essential for the diagnosis.

## Notes

### Competing interests

The authors declare that they have no competing interests.
